# Potential intermediate-term survival differences among heart transplant recipients from circulatory death vs brain death donors

**DOI:** 10.1016/j.jhlto.2025.100342

**Published:** 2025-07-10

**Authors:** Anh Nguyen, Abbas Rana, Alexis Shafii, Gabriel Loor, Andrew Civitello, Jose Euberto Mendez Reyes, O. Howard Frazier, Todd Rosengart, Kenneth Liao

**Affiliations:** a Division of Cardiothoracic Transplantation and Circulatory Support, Department of Surgery, Baylor College of Medicine, Houston, Texas; b Division of Abdominal Transplantation, Department of Surgery, Baylor College of Medicine, Houston, Texas; c Division of Cardiology, Department of Medicine, Baylor College of Medicine, Houston, Texas; d Cardiothoracic Surgery, Department of Surgery, Baylor College of Medicine, Houston, Texas

**Keywords:** heart transplant, survival, donation after circulatory death, donation after brain death, propensity-score matching

## Abstract

**Background:**

This study builds upon previous analyses by examining heart transplant survival from donation after circulatory death (DCD) vs donation after brain death (DBD) using the United Network for Organ Sharing (UNOS) database, with follow-up extended to 3 years post-transplant.

**Methods:**

We conducted a retrospective cohort study of 1,453 DCD and 16,561 DBD adult heart transplants from January 2019 to June 2024 using the UNOS database. Propensity scores were generated based on clinically relevant covariates, and 1-to-1 propensity-score matching was performed. Survival analysis was conducted using Cox proportional hazards regression and Kaplan-Meier curves, with the log-rank test comparing overall survival and the Wald test examining yearly survival rates between DCD and DBD groups.

**Results:**

Mortality was not significantly different between DCD and DBD total cohorts (hazard ratio [HR] = 1.1, 95% confidence interval [CI] 0.9-1.3, *p* = 0.493). After propensity-score matching, balanced cohorts of 1,423 DCD and 1,423 DBD transplants were created with standardized mean difference among covariates well below 6%. In the matched cohort, DCD transplant mortality was 1.2 times higher than that of DBD transplants (HR 1.2, 95% CI 0.9-1.5). Kaplan-Meier curves revealed nonsignificantly lower overall survival for DCD recipients (log-rank *p* = 0.096). Survival rates were comparable in year 1: 91.6% vs 91.5%, *p* = 0.96, but significant differences emerged in subsequent years: 84.7% vs 89.4%, *p* = 0.007 in year 2; 80.3% vs 85.6%, *p* = 0.025 in year 3.

**Conclusions:**

Intermediate-term survival following DCD heart transplantation may be lower compared to DBD transplantation. Further investigation is warranted to identify the underlying factors contributing to this potential disparity.

## Background

Heart failure is a leading global health challenge, affecting over 64 million people worldwide and more than 6.7 million adults in the United States annually.^1^ Heart transplantation is the definitive treatment for advanced heart failure, significantly extending survival and improving quality of life. However, the limited availability of donor hearts cannot meet the needs of the increasing number of patients on the waiting list.^2^ Without heart transplants, more than 30% waitlisted patients die within the first year.^3^ In addition to conventional donation after brain death (DBD), utilizing hearts from donation after circulatory death (DCD) can partially address this pressing need. It was estimated that the donor pool could be increased by up to 30% if DCD organs were used effectively.^4^ However, DCD heart transplantation has not been as widely implemented as other organs due to the concerns of warm ischemic injury to the donor heart and increased perioperative cardiac dysfunction.^5^, ^6^ A randomized clinical trial started in 2019 has published its findings in 2023 in New England Journal of Medicine stating that 6-month survival after DCD heart transplantation with the use of extracorporeal warm machine perfusion was not inferior to that after standard DBD heart transplantation with donor hearts preserved in cold storage.^7^ Another study using the United Network for Organ Sharing (UNOS) database from 2019-2021 published in 2023 in Journal of Thoracic and Cardiovascular Surgery also found that heart transplantation with DCD donors has comparable 6-month survival as compared to DBD transplants.^8^ A more recent study using UNOS data up to June 2022, published in 2024 in Journal of Heart and Lung Transplantation, demonstrated comparable survival rates at 2 years post-transplant between DCD (80%) and DBD (88%) heart transplants (*p* = 0.22).^9^ In this study, we accessed intermediate-term survival of DCD vs DBD heart transplantation by extending the analysis up to 3 years post-transplant.

## Methods

### Data source

We conducted a retrospective cohort study of adult heart transplants from January 2019 to June 2024 using the UNOS Standard Transplant Analysis and Research data. From the thoracic organ transplant recipient file, we identified 1,453 DCD and 16,561 DBD adult heart transplants from January 1, 2019, to June 30, 2024, after excluding recipients less than 18 years old, heart-lung transplants. Given the robustness of the large national dataset, we opted to exclude patients with missing survival follow-up data (3.8%) rather than perform imputations. This approach was chosen to maintain the integrity of our analysis and avoid potential biases introduced by imputation methods.^10^

### Statistical analysis

To address baseline differences between DCD and DBD cohorts, we performed 1-to-1 propensity-score matching using the nearest neighbor method without replacement. Propensity scores were generated based on clinically relevant covariates identified from the literature. Recipient covariates were age, ethnicity, gender mismatch, end listing status, obesity, diabetes, pretransplant use of inotropes, dialysis, ventilator, extracorporeal membrane oxygenation (ECMO), intra-aortic balloon pump (IABP), ventricular assist device (VAD), and transplant regions. Donor covariates were age, ethnicity, donor <80% recipient weight, hypertension, left ventricular function, diabetes, and travel distance.

Missing data among variables included in the propensity-score model were well below 2% so they were marked as unknown in the analysis. The proportion of missing data was as follows: recipient ethnicity (0.22%), recipient diabetes (0.07%), recipient pretransplant dialysis (0.19%), donor ethnicity (0.08%), donor hypertension (1.48%), donor diabetes (1.38%), and donor left ventricular ejection fraction (0.22%). After propensity-score matching, the balance of covariates was assessed by their standardized mean differences with the widely accepted threshold of less than 10%.^11^ Our covariate standardized mean differences were all less than 6% (Table 1), which were conservatively considered well balanced between DCD and DBD groups.

**Table 1:** Baseline Recipient and Donor Characteristics in DCD and DBD Heart Transplants

	All patients			Propensity-matched patients			
	DCD (*n* = 1,453)	DBD (*n* = 16,561)	*p*-value	DCD (*n* = 1,423)	DBD (*n* = 1,423)	*p*-value	SMD
*Recipient characteristics*							
Age (years), median (IQR)	57 (46-64)	57 (46-63)	0.171	57 (46-64)	56 (46-63)	0.165	5.6%
Ethnicity, N (%)			<0.001			0.309	−1.8%
White	931 (64.3)	9,608 (58.1)		916 (64.4)	915 (64.3)		
Black	332 (22.9)	4,253 (25.7)		327 (23.0)	333 (23.4)		
Hispanic	142 (9.8)	1,796 (10.9)		140 (9.8)	118 (8.3)		
Asian	31 (2.1)	645 (3.9)		30 (2.1)	40 (2.8)		
American Indian	6 (0.4)	74 (0.5)		5 (0.4)	6 (0.4)		
Pacific Islander	4 (0.3)	63 (0.4)		4 (0.3)	5 (0.4)		
Multiracial	1 (0.1)	88 (0.5)		1 (0.1)	6 (0.4)		
Gender mismatch, N (%)	225 (15.5)	3,375 (20.4)	<0.001	219 (15.4)	209 (14.7)	0.600	1.8%
End listing status, N (%)			<0.001			0.041	−3.0%
1	59 (4.1)	1,999 (12.1)		55 (3.9)	88 (6.2)		
2	556 (38.3)	8,683 (52.4)		545 (38.3)	492 (35.6)		
3	250 (17.2)	2,415 (14.6)		244 (17.2)	238 (16.7)		
4	387 (26.6)	2,638 (15.9)		380 (26.7)	385 (27.1)		
5	30 (2.1)	156 (0.9)		28 (2.0)	29 (2.0)		
6	171 (11.8)	670 (4.1)		171 (12.0)	191 (13.4)		
Obesity, N (%)	526 (36.2)	5,470 (33.0)	0.014	518 (36.4)	485 (34.1)	0.195	4.9%
Diabetes, N (%)	466 (32.1)	4,956 (29.9)	0.084	455 (32.0)	450 (31.6)	0.840	0.8%
PreTx inotropic support, N (%)	482 (33.2)	6,751 (40.8)	<0.001	477 (33.5)	462 (32.5)	0.550	2.2%
PreTx dialysis, N (%)	63 (4.4)	1,029 (6.2)	0.004	61 (4.3)	68 (4.8)	0.528	−2.2%
PreTx ventilator, N (%)	13 (0.9)	369 (2.2)	0.001	13 (0.9)	13 (0.9)	1.000	0.0%
PreTx ECMO, N (%)	38 (2.6)	1,102 (6.7)	<0.001	37 (2.6)	41 (2.9)	0.646	−1.3%
PreTx IABP, N (%)	208 (14.3)	4,475 (27.0)	<0.001	204 (14.3)	185 (13.0)	0.300	3.3%
PreTx VAD, N (%)	495 (34.1)	4,599 (27.8)	<0.001	484 (34.0)	494 (34.7)	0.693	−1.5%
Transplant region, N (%)			<0.001			<0.001	−1.6%
Region 1	190 (13.1)	788 (4.8)		187 (13.1)	61 (4.3)		
Region 2	49 (3.4)	1,592 (9.6)		48 (3.4)	89 (6.3)		
Region 3	126 (8.7)	1,941 (11.7)		122 (8.6)	153 (10.8)		
Region 4	78 (5.4)	1,539 (9.3)		75 (5.3)	115 (8.1)		
Region 5	250 (17.2)	2,774 (16.8)		240 (16.9)	209 (14.7)		
Region 6	36 (2.5)	492 (3.0)		35 (2.5)	69 (4.9)		
Region 7	87 (6.0)	1,509 (9.1)		87 (6.1)	128 (9.0)		
Region 8	89 (6.1)	1,061 (6.4)		88 (6.2)	103 (7.2)		
Region 9	120 (8.3)	1,313 (7.9)		116 (8.2)	111 (7.8)		
Region 10	34 (2.3)	1,305 (7.9)		34 (2.4)	139 (9.8)		
Region 11	394 (27.1)	2,247 (13.6)		391 (27.5)	246 (17.3)		

*Donor characteristics*							
Age (years), median (IQR)	31 (24-38)	32 (25-40)	0.559	31 (24-38)	30 (22-38)	0.353	0.0%
Ethnicity, N (%)			<0.001			0.002	−2.3%
White	1,105 (76.3)	10,143 (61.3)		1,085 (76.3)	1,017 (71.5)		
Black	137 (9.5)	2,767 (16.7)		136 (9.6)	205 (14.4)		
Hispanic	172 (11.9)	3,091 (18.7)		167 (11.7)	172 (12.1)		
Asian	17 (1.2)	291 (1.8)		17 (1.2)	17 (1.2)		
American Indian	12 (0.8)	143 (0.9)		12 (0.8)	10 (0.7)		
Pacific Islander	0 (0.0)	30 (0.2)		0 (0.0)	0 (0.0)		
Multiracial	6 (0.4)	85 (0.5)		6 (0.4)	2 (0.1)		
D/R weight <80%, N (%)	290 (20.0)	2,535 (15.3)	<0.001	286 (20.1)	293 (20.6)	0.744	−1.3%
Hypertension, N (%)	190 (13.2)	2,564 (15.7)	0.010	187 (13.1)	206 (14.5)	0.302	−3.8%
Diabetes, N (%)	47 (3.3)	672 (4.1)	0.107	45 (3.2)	40 (2.8)	0.582	1.9%
LV ejection fraction, N (%)	62 (59-66)	60 (56-65)	<0.001	62 (59-66)	62 (59-66)	0.913	−1.3%
Travel distance, median (IQR)	312 (128-543)	236 (100-407)	<0.001	315 (131-543)	311 (137-481)	0.276	4.7%

Abbreviations: DBD, donation after brain death; DCD, donation after circulatory death; D/R, donor/recipient; ECMO, extracorporeal membrane oxygenation; IABP, intra-aortic balloon pump; IQR, interquartile range; PreTx, pretransplant; LV, left ventricular; SMD, standardized mean difference; VAD, ventricular assist device.

Analyses were conducted for both the total cohorts and the propensity-score–matched cohorts. Patient characteristics were reported as frequencies and proportions for categorical variables and as median and interquartile range (IQR) for continuous variables. Wilcoxon rank sum test was used to compare continuous variables and Pearson chi-square test was used to compare categorical variables between DCD and BDB cohorts. Cox proportional hazards regression and Kaplan-Meier graph were used for survival analysis. We used the log-rank test to compare overall survival and the Wald test to examine survival rates by year between DCD and DBD groups at 1-, 2-, and 3-year follow-up. All tests were 2-tailed with alpha level of 0.05. Analyses were performed on Stata version 18.0 (Stata Corp LLC, College Station, TX).

This study was approved with a waiver of patient consent and Health Insurance Portability and Accountability Act authorization by the Institutional Review Board for Human Subject Research for Baylor College of Medicine and Affiliated Hospitals (H-51123, approved on November 29, 2022).

## Results

### Recipient characteristics

A total of 18,014 heart transplant patients were included in our study, with 1,453 (8.1%) being DCD recipients and 16,561 (91.9%) being DBD recipients. Both groups had the same median age: 57 years (IQR 46-64) for DCD and 57 years (IQR 46-63) for DBD, *p* = 0.171. The DCD cohort had a higher proportion of Whites (64.3% vs 58.1%), and fewer Blacks (22.9% vs 25.7%) and Hispanics (9.8% vs 10.9%), *p* < 0.001. DCD transplants were less likely to be gender mismatched (15.5% vs 20.4%, *p* < 0.001) compared to DBD transplants. DCD recipients were less likely to have end listing status 1 (4.1% vs 12.1%) or status 2 (38.3% vs 52.4%) and more likely to have end listing status 3 (17.2% vs 14.6%), status 4 (26.6% vs 15.9%), status 5 (2.1% vs 0.9%), or status 6 (11.8% vs 4.1%), *p* < 0.001. Before transplant, DCD candidates were more likely to be obese (36.2% vs 33.0%, *p* = 0.014) or on VAD (34.1% vs 27.8%, *p* < 0.001) but less likely to be on inotropic support (33.2% vs 40.8%, *p* < 0.001), dialysis (4.4% vs 6.2%, *p* = 0.004), ventilator (0.9% vs 2.2%, *p* = 0.001), ECMO (2.6% vs 6.7%, *p* < 0.001), or IABP (14.3% vs 27.0%, *p* < 0.001). After propensity-score matching, we had balanced cohorts of 1,423 DCD and 1,423 DBD recipients. In the matched cohorts, all these recipient characteristics were no longer significantly different between DCD and DBD groups (*p* > 0.05) except for end listing status and transplant regions. However, the standardized mean difference of these 2 factors among DCD and DBD groups was well below the threshold of 10% (3.0% and −1.6%, respectively). Details are presented in Table 1.

### Donor characteristics

DCD and DBD donors had similar median ages: 31 years (IQR 24-38) for DCD and 32 years (IQR 25-40) for DBD, *p* = 0.559. The DCD cohort had a higher proportion of Whites (76.3% vs 61.3%), and fewer Blacks (9.5% vs 16.7%) and Hispanics (11.9% vs 18.7%), *p* < 0.001. DCD transplants were more likely to have a donor/recipient weight ratio <80% (20.0% vs 15.3%, *p* < 0.001) compared to DBD transplants. DCD donors were less likely to have hypertension (13.2% vs 15.7%, *p* = 0.010) or diabetes (3.3% vs 4.1%, *p* = 0.107). The median left ventricular ejection fraction was significantly higher in DCD donors (62%, IQR 59%-66%) compared to DBD donors (60%, IQR 56%-65%), *p* < 0.001. DCD hearts also had longer travel distances than DBD ones (312 vs 236 miles, *p* < 0.001). In the matched cohorts, all these donor characteristics were no longer significantly different between DCD and DBD groups, except for ethnicity. However, the standardized mean difference for ethnicity between DCD and DBD cohorts was only −2.3%. Details are presented in Table 1.

### Survival of DCD and DBD heart transplants

**Figure 1:**
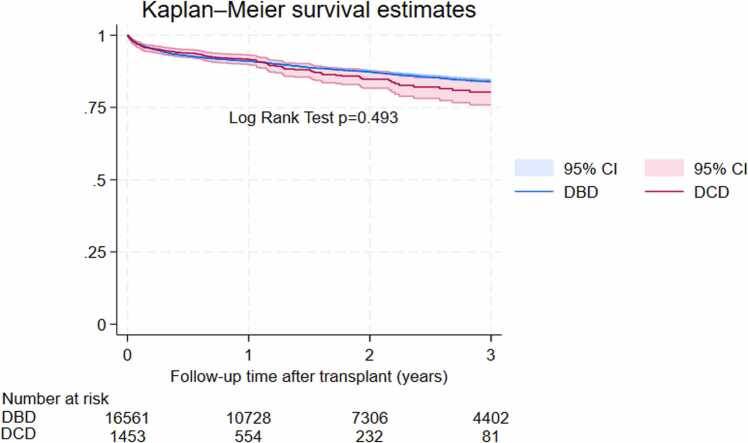
Total cohorts Kaplan-Meir curves comparing survival after DCD vs DBD heart transplants. DBD, donation after brain death; DCD, donation after circulatory death.

**Figure 2:**
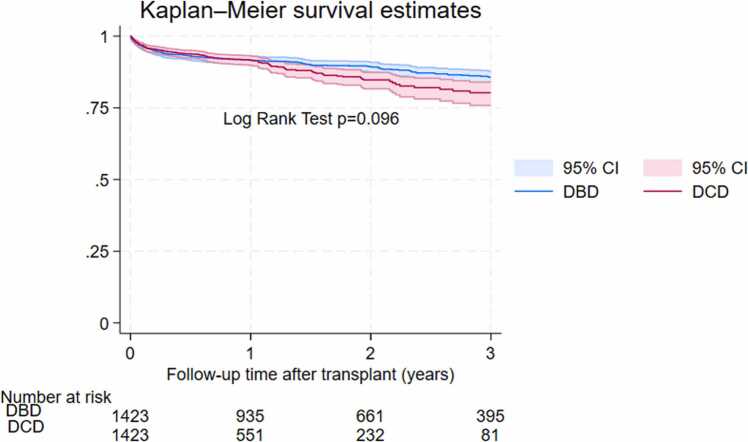
Matched cohorts Kaplan-Meir curves comparing survival after DCD vs DBD heart transplants. DBD, donation after brain death; DCD, donation after circulatory death.

Mortality was not significantly different between the total cohorts of 1,453 DCD and 16,561 DBD adult heart transplants (hazard ratio [HR] = 1.1, 95% confidence interval [CI] 0.9-1.3, *p* = 0.493). Though not statistically significant, in the matched cohort, DCD heart transplants showed a 20% increase in mortality compared to DBD transplants (HR 1.2, 95% CI 0.9-1.5, *p* = 0.096). Kaplan-Meier curves revealed nonsignificantly lower survival for DCD transplants compared to DBD transplants in the total cohorts (Figure 1) and in the matched cohorts (Figure 2). While survival rates of DCD and DBD groups were comparable in year 1 (91.6% vs 91.5%, *p* = 0.96), significant differences emerged in subsequent years: 84.7% vs 89.4%, *p* = 0.007 in year 2; 80.3% vs 85.6%, *p* = 0.025 in year 3. Details are presented in Table 2.

**Table 2:** Survival Rates After DCD vs DBD Heart Transplants

Years post-transplant	Unmatched cohort survival % (95% CI)			Matched cohort survival % (95% CI)		
	DCD (*n* = 1,453)	DBD (*n* = 16,561)	*p*-value	DCD (*n* = 1,423)	DBD (*n* = 1,423)	*p*-value
1	91.6 (89.8-93.1)	91.0 (90.5-91.4)	0.493 HR = 1.1 (0.9-1.3)	91.6 (90.0-93.2)	91.5 (90.0-92.9)	0.096 HR = 1.2 (0.9-1.5)
2	84.8 (81.7-87.4)	87.3 (86.7-87.8)		84.7 (81.6-87.4)	89.4 (87.5-90.1)	
3	80.3 (75.9-84.0)	83.8 (83.1-84.5)		80.3 (75.8-84.0)	85.6 (83.2-87.7)	

Abbreviations: CI, confidence interval; DBD, donation after brain death; DCD, donation after circulatory death; HR, hazard ratio.

## Discussion

Due to the shortage of donated organs, DCD donors have been utilized to expand the donor pool alongside DBD donors. However, the utilization of DCD hearts in the United States has lagged behind other organs such as the liver, kidney, and lung.^12^, ^13^, ^14^, ^15^ Concerns about warm ischemic injury and the ethical and technical challenges of assessing asystole DCD donor hearts after circulatory death have been major barriers.^16^, ^17^ Recent improvements in ex vivo warm perfusion system and normothermic regional perfusion technologies have made it possible to recondition and assess DCD hearts for transplantation suitability, pacing the way forward for DCD heart transplantation.^18^ Initial success of DCD heart transplants was reported in Australia and the United Kingdom.^19^, ^20^, ^21^, ^22^ They found that DCD heart transplantation had comparable survival to DBD ones. These early findings were encouraging but limited to retrospective case reports or case series of at most 79 DCD heart transplants at single centers. There has been a randomized controlled trial at 15 centers in the United States to assess outcomes of 90 DCD vs 90 DBD heart transplants. This trial concluded that 6-month survival after transplantation with DCD hearts with the use of extracorporeal machine warm perfusion was not inferior to that of DBD hearts with the use of cold storage.^7^ A large single-center study in the United States with 122 DCD transplants concluded that outcomes among DCD recipients are noninferior to those of DBD recipients within the first year after transplant.^23^ There are also other publications using UNOS registry data with up to 425 DCD heart transplants, followed up to 2 years.^8^, ^9^, ^24^ These studies showed similar short-term survival of DCD recipients as compared to DBD ones. Our study adds to the existing literature by including a larger cohort size of 1,453 DCD and 16,561 heart transplants with a longer post-transplant follow-up term up to 3 years. Consistent with other previous studies, we also found no significant difference in 1-year survival between DCD and DBD heart transplants (91.6% vs 91.5%, *p* = 0.96). However, significant differences emerged in subsequent years: 84.7% vs 89.4%, *p* = 0.007 in year 2; 80.3% vs 85.6%, *p* = 0.025 in year 3, respectively. A previous study using UNOS registry reported a lower 2-year survival for DCD (80%) as compared to DBD recipients (88%) but the difference did not reach statistical significance (*p* = 0.22) given the smaller sample size of 425 DCD and 850 DBD heart transplants as compared to ours.^9^ A multicenter retrospective observational study from 15 major transplant centers in the UK, Spain, United States, and Belgium with 157 DCD heart transplants reported similar survival between DCD and DBD in the first 5 years but looking at their Kaplan-Meier graphs in Figure 2, DCD line crossed and went under DBD line after year 6.^25^ To our knowledge, our study is the largest analysis of DCD heart transplant survival published to date. We will need to wait for the report of the intermediate-term survival of the randomized controlled clinical trial (NCT03831048) to make comparison. However, our study should be more robust since it recruited a real-world all-inclusive cohort of patients in the trial as well as outside of the trial. Our study included DCD hearts retrieved with normothermic regional perfusion as well while the trial was only restricted to direct procurement with ex vivo machine perfusion.

### Study limitations

Our study is not a randomized controlled clinical trial, so it has certain limitations inherent to observational studies. The DCD patients in our study were generally healthier, with fewer having end listing status 1 (4.1% vs 12.1%) or status 2 (38.3% vs 52.4%), *p* < 0.001. Additionally, DCD candidates were less likely to be on inotropic support (33.2% vs 40.8%, *p* < 0.001), dialysis (4.4% vs 6.2%, *p* = 0.004), ventilator (0.9% vs 2.2%, *p* = 0.001), ECMO (2.6% vs 6.7%, *p* < 0.001), or IABP (14.3% vs 27.0%, *p* < 0.001). DCD recipients were also less likely to be gender mismatched (15.5% vs 20.4%, *p* < 0.001), more likely to be White (64.3% vs 58.1%), and less likely to be Black (22.9% vs 25.7%) or Hispanic (9.8% vs 10.9%), *p* < 0.001, all of which are known to be associated with better transplant outcomes.^26^, ^27^, ^28^ After propensity-score matching, however, all these recipient characteristics were no longer significantly different between DCD and DBD groups (standardized mean differences were well under 10%). However, there might be residual confounding from unmeasured confounders that were not included in our analysis and could only be addressed by a randomized controlled clinical trial.

The UNOS data did not include certain variables that might affect transplant outcomes, such as the time from withdrawal of life support to circulatory arrest and the time from asystole to reperfusion. The actual ischemic time for DCD hearts that did not experience true ischemia while on an ex vivo perfusion machine was also undetermined. Additionally, the techniques used to procure DCD hearts were not recorded in the UNOS registry, whether it was normothermic regional perfusion of donor hearts or direct procurement followed by ex vivo perfusion.^18^, ^24^ Further studies with more detailed data are needed to understand the impact of these factors on the quality of DCD hearts and to develop strategies to reduce heart tissue injury, thereby improving DCD heart transplant survival.^5^, ^6^, ^29^ Studies have shown DCD hearts had more profound changes in myocardial edema, inflammation, and injury than DBD hearts.^29^, ^30^ However, how these factors among others have more impact on patient survival later than earlier needs further research looking into the mechanisms. We had relatively small DCD numbers at risk at year 3 (81) and year 4 (11), and the Kaplan-Meir graph in Figure 2 showed a wide confidence interval. However, given the large effect size, the survival difference between DCD and DBD groups was still statistically significant and there was no overlapping between the 2 corresponding confidence intervals. As we have more DCD in the registry in the coming years, we should have more power to produce more reliable estimates. The learning curve for DCD hearts could potentially affect patient survival and need to be monitored over time across different centers with various levels of experience and expertise.

Our findings do not discourage the use of DCD hearts. They play a crucial role in expanding the donor pool and reducing waitlist time and mortality for specific groups of heart transplant candidates.^3^, ^9^, ^31^ On the contrary, we strongly recommend increased efforts and investment in studying risk factors and testing solutions to improve the survival of DCD heart transplants.

## Conclusions

In this study of the UNOS registry, we found that propensity-score–matched DCD heart transplants had lower survival rates from year 2 to year 3 post-transplant compared to DBD transplants. Future studies are needed to confirm this finding and to understand the mechanisms and dynamics of ischemic heart injury during DCD heart procurement to improve transplant outcomes in this population.

## Author contributions

**Anh Nguyen:** Conceptualization, Methodology, Formal analysis, Data curation, Writing – original draft. **Abbas Rana:** Methodology, Resources, Writing – review & editing. **Alexis Shafii:** Methodology, Resources, Writing – review & editing. **Gabriel Loor:** Methodology, Resources, Writing – review & editing. **Andrew Civitello:** Methodology, Resources, Writing – review & editing. **Jose Euberto Mendez Reyes:** Methodology, Validation, Writing – review & editing. **O. Howard Frazier:** Methodology, Resources, Writing – review & editing. **Todd Rosengart:** Resources, Writing – review & editing, Supervision. **Kenneth Liao:** Conceptualization, Methodology, Resources, Writing – review & editing, Supervision.

## Disclosure statement

The authors declare the following financial interests/personal relationships which may be considered as potential competing interests: O.H. Frazier reports a relationship with BiVACOR that includes board membership and travel reimbursement. The other authors declare that they have no known competing financial interests or personal relationships that could have appeared to influence the work reported in this paper.

This project does not have funding support from any sources.
